# Transarterial injection of recombinant human type-5 adenovirus H101 in combination with transarterial chemoembolization (TACE) improves overall and progressive-free survival in unresectable hepatocellular carcinoma (HCC)

**DOI:** 10.1186/s12885-015-1715-x

**Published:** 2015-10-15

**Authors:** Xiao-jun Lin, Qi-jiong Li, Xiang-ming Lao, Han Yang, Sheng-ping Li

**Affiliations:** 1 Department of Hepatobiliary Oncology, Sun Yat-Sen University Cancer Center, 651 Dongfeng Road East, Guangzhou, Guangdong 510060 China; 2 Department of Thoracic Surgery, Sun Yat-sen University Cancer Center, State Key Laboratory of Oncology in South China, Guangzhou, Guangdong, China

**Keywords:** Hepatocellular carcinoma (HCC), Transhepatic arterial chemoembolization (TACE), H101, Tumor response

## Abstract

**Background:**

The aim of this study was to determine the clinical benefit of transhepatic arterial chemoembolization (TACE) with or without recombinant human adenovirus type 5 (H101) administration for the treatment of patients with hepatocellular carcinoma (HCC).

**Methods:**

Tumor response, progression-free survival (PFS), and overall survival(OS) were retrospectively evaluated in consecutive patients with unresectable HCC who received TACE with or without H101 between April 2012 and April 2013.

**Results:**

Patients with unresectable HCC were treated with transarterial injection of H101 with TACE (H101 group, *n* = 87) or TACE alone (control group, *n* = 88). Clinicopathological features were similar between the groups. Treatment response was significantly different between the groups (*P* = 0.01). In the H101 group, 25 patients demonstrated a complete response (CR, 28.7 %); 28 patients, a partial response (PR, 32.2 %); 23 patients, stable disease (SD, 26.4 %); and 11 patients, progressive disease (PD, 12.6 %). In the control group, 13 patients demonstrated CR (14.8 %); 19, PR (21.6 %); 34, SD (38.6 %); and 22, PD (25 %). OS and PFS was also significantly different between the groups. In the H101 group, median OS and PFS were 12.8 and 10.49 months, whereas in the control group they were 11.6 and 9.72 months, respectively (OS: *P* = 0.046; PFS: *P* = 0.044).

**Conclusion:**

In patients with unresectable HCC, H101 combined with TACE improves OS, PFS and treatment response compared with TACE alone.

**Electronic supplementary material:**

The online version of this article (doi:10.1186/s12885-015-1715-x) contains supplementary material, which is available to authorized users.

## Background

Hepatocellular carcinoma (HCC) is the third most common cause of cancer-related death worldwide [1]. Less than 20 % of patients with HCC are eligible for potentially curative liver transplantation or surgical resection [2]. Worldwide, transhepatic arterial chemoembolization (TACE) is regarded as the best palliative treatment for unresectable HCC and has been shown to provide a clinical survival benefit [2], albeit with poor prognosis [3] suggesting that additional strategies are needed to improve patient prognosis.

Gene therapy, especially oncolytic viral therapy, is a promising treatment for liver tumors and is being increasingly used in the clinic with favorable results [4]. H101 is a recombinant human type-5 adenovirus (Ad5) in which the gene encoding the 55 kDa E1B protein responsible for p53-binding and inactivation has been deleted to confer p53-selective replication of oncolytic viruses inducing accumulation of p53 leading to direct and selective cytotoxicity in tumor cells during replication [5]. The H101 virus produced by Shanghai Sunway Biotech also contains a deletion of a 78.3–85.8 μm gene segment in the E3 region. The E3 region is responsible for the inhibition of host immunity, which enhances virus replication and spread in tumor cells [6].

Previous studies evaluating the safety of H101 as a direct injection [7] or transarterial infusion combined with TACE [8, 9], but the result were insufficient because of the small patient numbers (10,27,1), moreover, without a control group. While this large sample-sized study has enrolled 87 patients treated by H101 with a control group(*n* = 88), aimed to demonstrate the effect for unresectable HCCs. In the current study, treatment-related tumor response, overall survival(OS) and progression free survival (PFS) rates between H101 plus TACE and TACE alone were compared as the primary endpoints. The secondary endpoint included an assessment of treatment-related adverse events (AEs).

## Methods

### Patient selection

This retrospective study was approved by the ethics committee of Sun Yat-Sen University Cancer Center, and was performed in accordance with the Helsinki Declaration of 1975, as revised in 1983. From April 2012 to April 2013, 367 consecutive patients with unresectable HCC who underwent TACE, with or without transarterial injection of H101, at the Sun Yat-Sen University Cancer Center were enrolled. The diagnosis of HCC was based on non-invasive criteria according to the recommendation of the European Association for the Study of the Liver (EASL) and the European Organization for Research and Treatment of Cancer (EORTC) [10]. The definition of surgical unresectability was as follows: (1) Child-Pugh classification B; (2) ≥3 tumor nodules of any size; and (3) the inability to ensure adequate function of the postresection liver volume. Eligibility criteria included: (1) no previous treatment for HCC before TACE; (2) adequate hematological function (Child-Pugh A or Child-Pugh B); (3) adequate renal function (serum creatinine < 140 μmol/L, and serum blood urea nitrogen < the upper limit of normal). Exclusion criteria included: (1) previous resection or ablation before TACE, (2) prior bland embolization; and (3) if the patient had received therapy with more than one type of embolic agent or transcatheter therapy. Patients who met the criteria provided written informed consent for the study.

### Treatment procedures

For each modality, a uniform treatment protocol was followed. TACE was performed through the femoral artery with use of the Seldinger technique with local anesthesia as previously reported [11]. The chemotherapeutic agents were infused into the hepatic artery supplying the tumor(s). Conventional chemoembolization was performed by administering carboplatin 300 mg (Bristol-Myers Squibb, NY, USA). Thereafter, chemolipiodolization was performed using epirubicin 50 mg (Pharmorubicin, Pfizer, Wuxi, China), and mitomycin 6 mg (Zhejiang Hisun Pharmaceutical Co. Ltd., Taizhou, China) mixed with 5 mL of lipiodol (Lipiodol Ultra-Fluide; Andre Guerbet Laboratories, France).

H101 was administered via the catheter into the hepatic artery supplying the tumor(s). A total of 1.0 × 10^12^ virus particles in 10 mL 0.9 % sodium chloride were administered. Sterile purified viral lots were produced for human clinical use by Shanghai Sunway Biotech (Shanghai, China), and tested for the titer, sterility, and general safety by the National Institute for the Control of Pharmaceutical and Biological Products (Beijing, China).

### Follow-up

Antitumor efficacy was evaluated by computed tomography/magnetic resonance imaging (CT/MRI) at 1 month post-treatment and every 3–4 months thereafter. Further treatments were based on clinical evaluation, laboratory values, and imaging response. Tumor response according to the modified Response Evaluation Criteria in Solid Tumors (mRECIST) guidelines [12] was independently assessed in a blinded manner by 3 qualified radiologists. When a difference of opinion occurred, a consensus was obtained through discussion.

Liver function tests, ascites, and encephalopathy were monitored during follow-up visits to assess for liver failure. Clinical AEs were graded according to the National Cancer Institute (NCI) Common Terminology Criteria for Adverse Events (CTCAE) v4.0 criteria [13].

### Statistical analysis

The statistical significance of the difference between the means of continuous variables was determined using the independent t-test. A P-value of 0.05 was considered to be statistically significant. The chi-squared test was used to compare categorical variables. The Kaplan–Meier method was used to estimate OS and PFS.

## Results

Patient demographics and clinical characteristics were similar between the groups and are shown in Table 1. From April 2012 to April 2013, 187 patients with unresectable HCC were treated with TACE plus H101 met the inclusion criteria (H101 group) (Additional file 1: Figure S1) . In the same period, 88 patients with unresectable HCC underwent conventional TACE alone met the inclusion criteria (control group).

**Table 1:** Patient demographics and characteristics

	Overall	H101	Control	*P*-Value	Overall Survival(%)		Median survival(mo)	Univariate	Multivariate	
					1-yr	2-yr		*P*-Value	ExpB (Hazard Ratio ,95 % CI)	*P*-Value
Gender				0.305				0.302		
Male	159	81	78		67	52	13.0			
Female	16	6	10		60	30	11.2			
Age				0.948				0.100		
Median	55.0	55.0	54.5							
< 60	114	59	55		68	49	12.5			
≥ 60	61	28	33		68	56	13.0			
Alpha-foetoprotein(ng/ml)				0.316						
	307.2	269.1	307.2							
Alpha-foetoprotein(ng/ml)				0.947				0.06	1.669(1.178–2.366)	0.004
≤ 20	53(30.1 %)	27(30.7)	26(29.5)		89	81	17.6			
20–400	42(23.9 %)	20(22.7)	22(25.0)		60	45	13.9			
≥ 400	80(45.5 %)	40(45.5)	40(45.5)		52	32	9.1			
Child Pugh grade				0.820				0.007	2.132(1.138–3.995)	0.018
A	154(88.0 %)	76(87.3 %)	78(88.6 %)		70	55	13.3			
B	21(12.0 %)	11(12.6)	10(11.3)		38	25	7.7			
ALB(g/L)				0.228				0.412		
Median	40.0	39.6	40.2							
≥ 35	131	61	70	0.166	62	50	11.7			
< 35	44	26	18		67	52	13.3			
Tbil(U/L)				0.386				0.003		
Median	16.4	16.1	16.8							
< 20	119	61	58	0.628	75	56	13.7			
≥ 20	56	26	30		48	40	8.0			
Virus infection				0.970				0.101		
none	15	7	8		88	66	13.6			
HBV	158	79	79		63	51	12.6			
HCV	2	1	1		50	0	13.9			
Platelet count (10E9/L)				0.630				0.676		
Median	167.0	179.2	154.5							
< 100	31	11	20	0.112	63	46	12.2		0.330(0.141–0.773)	0.011
≥ 100	144	76	68		67	53	12.8			
No. of tumours				1.000				<0.001	2.024(1.127–3.633)	0.018
≤ 3	127	69	70		74	57	13.7			
> 3	36	18	18		38	30	7.6			
Tumour size (cm)				0.730				0.028	2.936(1.297–6.650)	0.010
≤ 5	45	21	24		91	75	18.1			
> 5	130	66	64		56	42	9.8			
Anti-HBV therapy				0.197				0.424		
Yes	56	32	24		60	54	13.3			
No	119	55	64		69	51	9.65			
H101				0.046				0.042	0.593(0.353–0.995)	0.048
Yes	87	87	0		69	60	12.8			
No	88	0	88		60	44	11.6			
BCLC stage				0.453				0.001	2.168(1.322–3.557)	0.002
A3	1	0	1							
A4	17	10	7		94	94	17.0			
B	108	50	58		72	56	12.96			
C	49	27	22		52	23	6.96			

*AFP* alpha fetoprotein, *ALB* serum albumin, *HBV* hepatitis B virus, *HCV* hepatitis C virus, *TBIL* total bilirubin, *PVT* portal vein thrombosis

Tumor response is shown in Table 2 significant difference was noted in tumor response between the two groups (Table 2). Furthermore, subgroup analysis according to treatment response showed that the number of each response type was significantly different between the groups (Table 2). In general, patients in the H101 group responded better to treatment compared with those who received TACE alone.

**Table 2:** Treatment response of H101 group and control group

	Overall	H101(none + Anti-HBV Therapy,*P* = 0.162)	Control	*P* value
				0.010
CR	38(21.7 %)	25 (28.7 %)(16 + 9)	13(14.8 %)	0.017
PR	47(26.9 %)	28(32.2 %)(21 + 7)	19(21.6 %)	0.172
SD	57(32.6 %)	23(26.4 %)(14 + 9)	34(38.6 %)	0.107
PD	33(18.9 %)	11(12.6 %)(4 + 7)	22(25 %)	0.011

All patients enrolled in H101 group were screened to sort out cases with anti-HBV therapy or without anti-HBV therapy.

None: Patients treated by H101 without anti-HBV therapy.

Anti-HBV Therapy: Patients treated by H101 with anti-HBV therapy session.

The majority of the patients (90.2 %) tested positive for hepatitis B virus (HBV) and some patients received anti-HBV agents that could potentially confound the beneficial effects of H101 as antiviral agents. To determine the effect of anti-HBV treatment on H101, patients were stratified by anti-HBV therapy administration. As shown in Table 2B, there was no significant difference in treatment response between the two subgroups.

Significant positive correlations have been reported between lipiodol accumulation observed on CT images and necrosis in resected tumors examined after TACE, and, therefore, intratumor lipiodol accumulation is regarded as an indicator of necrosis [14, 15]. The degree of lipiodol retention for all patients is presented in Table 3. There were significant differences in lipiodol accumulation between the two treatment groups (*P* = 0.002).

**Table 3:** Tumor response

	Overall	H101	Control	*P* value
Alpha-fetoprotein(ng/ml) reduce				0.448
≥ 20 %	77	41	36	
< 20 %	98	46	52	
Lipiodol retention				0.002
None	20	7	13	
Partial	132	61	71	
Complete	23	19	4	

*CR* complete response, *PR* partial response, *SD* stable disease, *PD* progressive disease

Blood samples for laboratory analysis were collected before and 1–2 days after TACE for each patient (Table 4). There were no significant differences in clinical parameters between the two groups with the exception of a significant increase in white blood cell count in the H101 group compared with the control group (*P* = 0.001). Post-treatment AEs are shown in Table 4 Fever was significantly higher in the H101 group compared with the control group (*P* = 0.023). No grade 4 clinical toxicity or procedure-related deaths (30 days) due to liver failure were experienced in either group. There were no major complications or grade 3–4 liver toxicities within the first post-treatment month. The overall frequency of treatment-emergent AEs was not significantly different between the groups (*P* = 0.263).

**Table 4:** Clinical adverse effects

	Overall	H101	Control	*P* value
Fever				0.023
> 38.5 °C	55.4 %	64.4 %	46.6 %	
≤ 38.5 °C	44.6 %	35.6 %	53.4 %	
Pain				0.875
Yes	65.1 %	64.4 %	65.9 %	
No	34.9 %	35.6 %	34.1 %	
Ascites				0.864
Yes	25.7 %	26.4 %	25 %	
No	74.3 %	73.6 %	75 %	
Acute renal failure				
Yes	5.1 %	5.7 %	4.5 %	0.896
No	94.9 %	94.3 %	95.5 %	
Encephalopathy				
Yes	0	0	0	
No	100 %	100 %	100 %	
White Blood Cell				
Before TACE	5.7	6.0	5.5	0.369
After TACE	7.64	7.0	8.84	0.991
Elevation	1.61	0.5	2.97	0.001
PLT				
Before	167.0	179.2	154.5	0.186
After	113.0	122.8	106.1	0.258
Elevation	49.0	52.7	48.4	0.480
ALT				
Before	41.5	43.2	41.4	0.371
After	167.1	153.0	200.9	0.405
Elevation	103.1	88.1	118.4	0.480
AST				
Before	50.9	57.1	46.4	0.249
After	221.3	225.4	213.5	0.993
Elevation	154.2	141.2	162.0	0.863
TBIL				
Before	16.3	16.1	16.8	0.657
After	30.4	28.3	31.8	0.162
Elevation	12.9	12.4	13.25	0.413
ALB				
Before	40.0	38.2	41.2	0.161
After	35.4	34.1	37.0	0.314
Elevation	4.1	3.6	4.4	0.226

*PLT* platelet count, *ALB* albumin, *ALT* alanine aminotransferase, *AST* aspartate aminotransferase, *PLT* platelet, *TACE* transhepatic arterial chemoembolization, *TBIL* total bilirubin

**Fig. 1:**
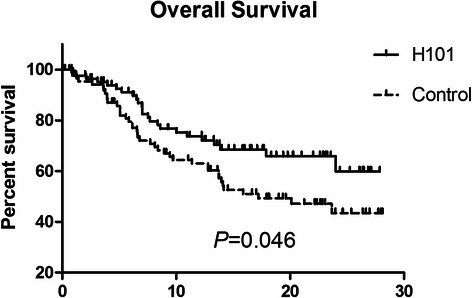
The Kaplan–Meier survival analysis comparing the overall survival from the first transcatheter therapy of advanced stage HCC patients who underwent TACE combined with H101 (H101 group, *n* = 87) and TACE alone (control group, *n* = 88).

The median OS time during follow-up was 12.8 months (mean ± SD: 12.95 ± 8.36 months) in the H101 group and 11.6 months (mean ± SD: 12.87 ± 8.28 months) in the control group. 24 patients (27.6 %) in the H101 group and 41 (46.6 %) in the control group expired. The causes of death included liver disease progression (46/65, 70.8 %), upper gastrointestinal hemorrhage (7/65, 10.8 %), encephalopathy (7/65, 10.8 %), and peritonitis and pneumonia (6/65, 9.2 %). There were no treatment-related deaths. The cumulative OS rates at 1 and 2 years were significantly different and were 69 and 60 % in the H101 group, respectively, 60 and 44 % in the control group, respectively (*P* = 0.046, Fig. 1). Univariate analysis by Cox-regression revealed 6 prognostic factors affecting OS: Child-Pugh grade (grade A vs. grade B, *P* = 0.007), total bilirubin (<20 vs. ≥20 U/L, *P* = 0.003), BCLC(Barcelona Clinic Liver Cancer )stage (*P* = 0.001), tumor number (≤3 vs. >3, *P* < 0.001), tumor size (≤5 cm vs. >5 cm, *P* = 0.028), and H101 (*P* = 0.042). Multivariate analysis by cox-regression revealed that AFP (*P* = 0.004), CHILD PUGH grade (*P* = 0.018), BCLC stage(*P* = 0.002), Platelet count (PLT)(*P* = 0.011), the number of tumors (*P* = 0.018), tumor size(*P* = 0.010)and H101 (*P* = 0.048) were independent prognostic factors of OS.

**Fig. 2:**
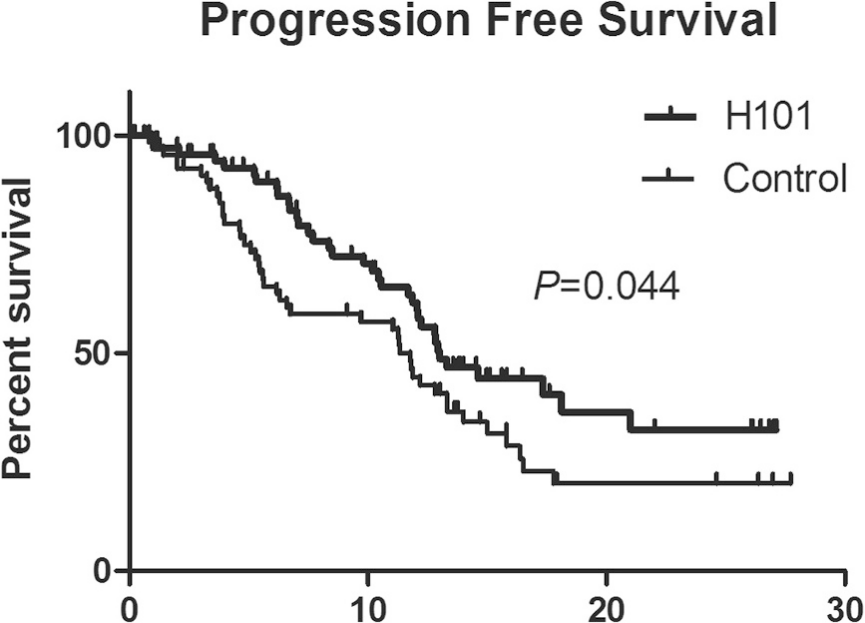
The Kaplan–Meier survival analysis for progression-free survival of the 74 patients with unresectable HCC who underwent TACE combined with H101 and the 68 patients with unresectable HCC who underwent TACE alone.

After the first post-treatment review, all 175 patients were assigned as CR, PR, SD, or PD according to mRECIST criteria. In total, 142 patients in both groups (H101: 74; control: 68) were judged as CR, PR, or SD. During follow-up, progression free survival was observed in these 142 patients. The median time to progression for the H101 and control groups were significantly different at 10.49 and 9.72 months, respectively (*P* = 0.044, Fig. 2).

In univariate analysis by cox-regression, 3 prognostic factors affecting tumor progression were identified: tumor number (*P* = 0.002), tumor size (*P* = 0.041), and treatment modality (H101; *P* = 0.036; Table 5). Multivariate analysis identified 4 prognostic factors as independent predictors of progression: the number of tumors (*P* = 0.001), tumor size (*P* = 0.041), Child-Pugh grade (*P* = 0.050) and treatment modality (H101) (*P* = 0.017, Table 5).

**Table 5:** Univariate and Multivariate analysis of PFS

	Cases				Univariate	Multivariate	
		1-yr survival rate(%)	2-yr survival rate(%)	Median survival(mo)	*P*-Value	ExpB (Hazard Ratio ,95 % CI)	*P*-Value
Gender							
Male	130	47	26	10.79	0.331		
Female	12	37	0	6.11			
Age							
< 60	92	49	29	10.49	0.180		
≥ 60	50	40	21	9.35			
Alpha-foetoprotein(ng/ml)							
≤ 20	42	50	44	11.99	0.445		
20–400	37	35	8	7.03			
≥ 400	63	51	28	8.37			
Child Pugh grade					0.047	2.852(1.002–8.293)	0.050
A	133	48	28	10.56			
B	9	22	0	7.07			
ALB(g/L)					0.307		
≥ 35	108	46	20	10.49			
< 35	34	47	47	10.25			
Tbil(U/L)					0.429		
< 20	104	49	23	11.18			
≥ 20	38	37	32	6.21			
Platelet count (10E9/L)							
< 100	19	74	32	13.57	0.144		
≥ 100	123	42	25	9.83			
No. of tumours					0.034	3.992(1.978–8.057)	0.001
≤ 3	118	51	28	11.04			
> 3	24	21	14	7.6			
Tumour size (cm)					0.988	2.667(1.041–6.832)	0.041
≤ 5	35	39	24	11.37			
> 5	107	48	25	8.37			
Virus infection					0.079		
none	14	59	59	13.05			
HBV	126	43	24	10.25			
HCV	2	50	0	11.30			
Anti-virus therapy					0.951		
Yes	33	44	30	8.37			
No	95	43	22	10.56			
H101					0.051	0.461(0.244–0.870)	0.017
Yes	74	51	32	10.49			
No	68	41	20	9.72			

## Discussion

The main purpose of this study was to compare the outcomes of patients with late stage HCC treated with two different methods of chemoembolization: a conventional method with commonly used protocols, and one using H101 virus. The data revealed that transcatheter therapy with H101 provided a significant tumor response and survival advantage over treatment with conventional chemoembolization (TACE alone) in patients with unresectable HCC.

H101 is an E1B-55 K-/E3B-deleted adenovirus [16], which has been used as an anticancer agent with the goal of restricting replication to p53-mutated neoplasm, sparing p53 wild-type human tissues. Preclinical studies have confirmed the anticancer activity of the H101 virus [17]. Clinical studies demonstrated the tolerability and anti-tumor efficacy of this agent as a monotherapy in patients with head and neck cancer [18, 19]. Different studies have compared the efficacy and safety of multiple routes of H101 administration in patients with HCC or liver tumors including hepatic arterial administration [20, 21],intravenous injection, and ultrasound-guided intratumoral injection [7, 22, 23]. Overall, H101 was safe when administered intratumorally, intraperitoneally, intraarterially, or intravenously at doses up to 3 × 10^11^ pfu [8, 24].

In this study, a significant difference in response rate was noted between the H101 and control groups. Radiologically, tumor response as determined by mRECIST was shown as obvious volume shrinkage and large areas of necrosis in tumor. The response rate of the control group was similar to that reported in previous studies of our department [25]. In the H101 group, greater improvements were seen especially with regard to CR and PD which may suggest more complete necrosis in the tumor and less lost-control. The mechanism behind the increased efficacy of H101 is not clear but may suggested as follows: 1)H101 is a p53-mutated specific agent, and up to 30–50 % [26] HCCs were found mutated or lost of p53. 2) Pei et al. [27] showed that HCC cells expressed high levels of inhibitor of apoptosis proteins, and were resistant to tumor necrosis factor (TNF)-related apoptosis while E1B-55 K-deleted oncolytic adenovirus showed partial antitumoural efficacy in the BEL7404 xenograft tumour model. 3)H101 has synergistic effect while combined with chemotherapy, and the enhanced antitumor effect was demonstrated in Hep3B (p53-null) and HepG2 (p53-wt) in vitro and in vivo [28].

The OS and PFS rate was significantly different between the two treatment modalities, and results from cox-regression showed H101 were the independent prognostic factor for these late stage HCC patients. These results coordinate with the response advantage of H101, demonstrate the survival advantage for HCCs. However, as generally accepted, beside tumor burden, overall survival in unresectable HCCs is affected by multiple reasons. First, OS in patients with HCC is greatly affected by the degree of liver dysfunction, and patients with Child-Pugh B liver function usually have poor survival regardless of the treatment regimen [29]. In many cases, liver function did not reflect the tumor response and in some patients liver function actually worsened with tumor shrinkage. In this study, most patients had a liver function status graded as Child-Pugh A(88 %), 21 Child-Pugh B cases were nearly even in the two groups(10:11), the bias to overall survival was insignificant. Second, TACE was the initial treatment for these patients, most of whom received subsequent treatments including resection, ablation, repeated TACE, and systemic therapies, or best supportive care. As our previous prospective clinical trial demonstrated, subsequent treatments can influence OS, especially for patients with large and multiple HCC at diagnosis; surgical resection for patients who responded well to TACE significantly prolonged survival, when compared to those who refused surgery [30]. In this study, patients whose tumor was downstaged were offered radical treatment including 36 patients for surgical resection and 29 for local ablation, most of which was CR, PR and some of SD, but without PD patient. These subsequent treatments most likely improved OS, which would enhance the advantage of tumor response.

More than 80 % of patients with HCC in Asia are hepatitis B virus positive, and most are receiving anti-viral therapy [31]. This could confound the results of any evaluation of H101 because it is a recombinant adenovirus and anti-viral therapy has the potential to prevent H101 replication. However, to the best of our knowledge, this has not been previously investigated. The most employed anti-viral agents for hepatitis B in our patients were lamivudine (35.5 %), adefovir dipivoxil (14.7 %), and entecavir (42.3 %). There are no reported studies demonstrating any potential interaction of these agents with adenovirus. Moreover, stratification of our patient data to those receiving anti-viral therapy or not, did not reveal any significant effect of antiviral therapy on H101 efficacy in terms of tumor response or OS and PFS.

Other than efficacy, safety and adverse events are important aspects to consider in patients undergoing viral therapy. The first case of a patient dying as a result of gene therapy was reported in 1999 by Marshall [32]. The patient, a relatively fit 18-year-old male with an inherited enzyme deficiency, received a dose of 4 × 10^13^ pfu of a replication-deficient adenovirus expressing the ornithine transcarbamylase gene. Less than 24 h later, he experienced hyperammonemia, acute respiratory distress syndrome, disseminated intravascular coagulation, and suffered multiorgan system failure. He died 4 days later, which questioned the safety of adenovirus for gene therapy [33, 34]. However, subsequent studies have found no mortality associated with adenoviral vector therapy and any complications are usually mild and reversible [8, 35], suggesting that the case reported by Marshall et al., may be a sporadic case of accidental death. In this study, no patients died and all AEs were reversible. The complication rates between H101 group and TACE alone (control) group were similar. Child-Pugh class A and B patients did not experience any major complications after treatment with H101, but did experience liver failure after treatment, but there was no statistically significant difference in liver toxicity at 1–2 months between the treatment groups. Increases in liver enzymes and total bilirubin levels and decreases in serum albumin levels were mild and not significantly different between the treatment groups. However, frequent high fever (*P* = 0.023) and an increase in the white blood cell count (*P* = 0.001) were apparent in the H101 group, which might be explained by the immune activation. Previous studies have noted an increase in inflammatory cytokine generation and fever after hepatic arterial infusion of adenovirus [36]. Interestingly, Lu et al., [36] found that during H101 injection, the efficacy was significantly higher in those who had fever than that in those who did not, suggesting that virus infection may activate the host immune system and the elevated cell-mediated immunity may play a role in the tumor regression. In this study, subgroup analysis based on fever did not reveal any differences between fever and treatment efficacy (OS, *P* = 0.109; PFS, *P* = 0.221).

This study has several limitations including its retrospective nature. As a case-controlled study, the survival benefit demonstrated must be considered preliminary and further prospective, randomized-controlled, long-term studies are needed to confirm our results.

## Conclusion

Transcatheter H101 therapy in combination with TACE for patients with unresectable HCC may provide a survival(OS and PFS) and tumor response advantage over treatment with conventional TACE alone.

## Additional file

Additional file 1: Figure S1. Flow diagram and randomization of study population. (DOCX 15 kb)

## Abbreviations

TACE: Transarterial chemoembolization
HCC: Hepatocellular carcinoma
H101: Recombinant human adenovirus type 5
PFS: Progression-free survival
OS: Overall survival
CR: Complete response
PR: Partial response
SD: Stable disease
PD: Progressive disease
AEs: Adverse events
EASL: European Association for the Study of the Liver
EORTC: European Organization for research and treatment of cancer
CT/MRI: Computed tomography/magnetic resonance imaging
mRECIST: Modified response evaluation criteria in solid tumors
NCI: National cancer institute
CTCAE: Common terminology criteria for adverse events
HBV: hepatitis B virus
BCLC: Barcelona clinic liver cancer
PLT: Platelet count
