# Supplementary material for: Transarterial injection of recombinant human type-5 adenovirus H101 in combination with transarterial chemoembolization (TACE) improves overall and progressive-free survival in unresectable hepatocellular carcinoma (HCC)
Source: BMC Cancer. 2015 Oct 15;15:707. doi: 10.1186/s12885-015-1715-x (PMC4608280; doi:10.1186/s12885-015-1715-x)
Supplement: Additional file 1: Figure S1. — Flow diagram and randomization of study population. (DOCX 15 kb) [file 12885_2015_1715_MOESM1_ESM.docx]

Flow diagram and randomization of patients with unresectable HCC, which was considered suitable for TACE.
